# Supplementary material for: Factors Contributing to the Comprehensive Use of Food Labels in Jordan
Source: Nutrients. 2023 Nov 23;15(23):4893. doi: 10.3390/nu15234893 (PMC10708443; doi:10.3390/nu15234893)
Supplement: Supplementary file 1 [file nutrients-15-04893-s001.zip › nutrients-2679556-supplementary.pdf]

Supplementary S1: Results of the factor analyses for attitude and practice scales.

Practice related to food labeling utilization.

An exploratory factor analysis was conducted on the 14-item 5-point Likert scale to understand practice of consumers in relation to food labels. The Kaiser-Meyer-Olkin (KMO) measure verified the sampling adequacy for the analysis, KMO = [0.932]. Bartlett's test of sphericity $\chi^2(df) = 7888.25(91)$, $p < 0.001$, indicated that correlations between items were sufficiently large for EFA.

Principal axis factoring was used to extract the underlying factors. Two factors were identified based on eigenvalues greater than one, supported by the scree plot. A varimax rotation was performed to aid in the interpretation of these two factors. Factor 1, labeled as "nutritional facts", primarily concerns nutritional values, ingredients, allergy information. Factor 2, "product information", primarily encapsulates items related to product name, country, expiration date. Further details are in Table S1. Together, the two factors explain [0.9930]% of the total variance in the responses. The Cronbach's alpha for the overall scale was [0.9292], indicating "good reliability", which was 0.8336 for Factor 1 and 0.9168 for Factor 2.

Table S1, Practice scale factor loading using varimax rotation method

**Question (item)**	**Factor 1**	**Factor 2**	**Uniqueness**
**Product info**			
P1-In the past six months, how many times have you read the product name on the label?	0.4901	**0.3828**	0.6133
P2-In the past six months, how many times have you read the country of origin on the label	0.4190	**0.5182**	0.5559
P3-In the past six months, how many times have you read the "best used by" a specific date on the label?	0.2020	**0.7664**	0.3718
P4-In the past six months, how many times have you read "best used until a specific date" on the label?	0.2217	**0.7323**	0.4146
P5-In the past six months, how many times have you read the preparation method on the label?	0.4543	**0.4519**	0.5895
P6-In the past six months, how many times have you read the storage method on the label?	0.5383	**0.4985**	0.4616
**Nutritional facts**			
P7-In the past six months, how many times have you read the serving information on the label?	**0.6539**	0.3082	0.4775
P8- In the past six months, how many times have you read the product name/food item on the label?	**0.5581**	0.4725	0.4653
P9- In the past six months, how many times have you read the nutritional claims (like low fat, heart-healthy) on the label?	**0.7022**	0.2629	0.4378
P10- In the past six months, how many times have you read the nutritional information on the label?	**0.7319**	0.3377	0.3503
P11- In the past six months, how many times have you read allergy information (like nut-free) on the label?	**0.7525**	0.2388	0.3767
P12-In the past six months, how many times have you read information about the ingredients (ingredients, quantity of ingredients) on the label?	**0.7130**	0.3262	0.3853
P13- In the past six months, how many times have you read information on the label about ingredients if they are genetically modified?	**0.7773**	0.1709	0.3666
P14- In the past six months, how many times have you read information on the label about ingredients if they are organic?	**0.7736**	0.1916	0.3649

Attitude related to food labeling utilization.

An exploratory factor analysis was conducted on the eight items using a 5-point Likert scale. The Kaiser-Meyer-Olkin (KMO) measure verified the sampling adequacy for the analysis, KMO = [0.837]. Bartlett's test of sphericity $\chi^2(df) = 2293.09(28)$, $p < 0.001$, indicated that correlations between items were sufficiently large for EFA. Principal axis factoring was used to extract the underlying factors. Two factors were identified based on eigenvalues (Factor 1: 6.90329; Factor 2: 0.86582), supported by the scree plot (Figure S1). A varimax rotation was performed to aid in the interpretation of these factors. Factor 1, which can be termed as "nutritional values", primarily encapsulates items related to “fat and sugar”. Factor 2, labeled as "label information", primarily concerns [using and characteristics of food labels. Together, the two factors explain 57.74% of the total variance in the responses. The Cronbach’s alpha for the overall scale was 0.8055 (0.8149 for Factor 1 and 6844 for Factor 2) indicating good reliability.

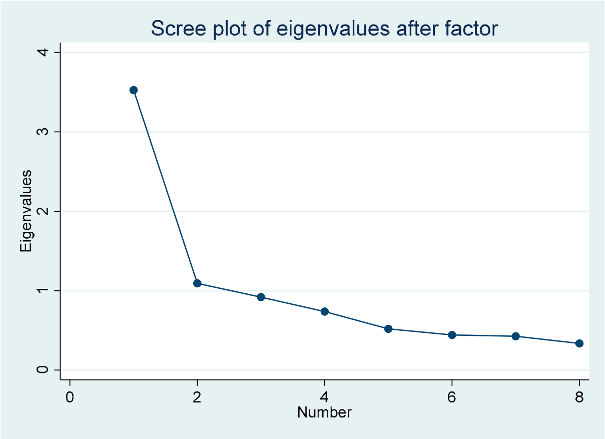

Figure S1, scree plot of eigenvalues for practice scale

Table S2, attitude scale factor loading using varimax method

**Question (item)**	**Factor 1**	**Factor 2**	**Uniqueness**
I frequently read the food labeling	0.5218	**0.4506**	0.5247
I believe food labels are important.	0.2037	**0.5856**	0.6155
I believe food labels provide sufficient information	-0.0185	**0.7279**	0.4698
I find the information on food labels understandable.	0.2703	**0.6602**	0.4911
I am confident in my understanding of food labels	0.5595	**0.5526**	0.3816
Reading the type and percentage of fat on food labels is important to me.	**0.8425**	0.1318	0.2729
Knowing the type of added sugar in products matters to me.	**0.7935**	0.1775	0.7935
I find it important to check if a fat product is hydrogenated when reading food labels.	**0.8422**	0.0647	0.2865
